# Risk factors associated with nonsteroidal anti-inflammatory drugs (NSAIDs)-induced gastrointestinal bleeding resulting on people over 60 years old in Beijing

**DOI:** 10.1097/MD.0000000000010665

**Published:** 2018-05-04

**Authors:** Tian-Yu Chi, Hong-Ming Zhu, Mei Zhang

**Affiliations:** Department of Gastroenterology, Xuanwu Hospital, Capital Medical University, Beijing, China.

**Keywords:** adverse drug reaction, elderly, gastrointestinal bleeding, nonsteroidal anti-inflammatory drugs, risk factors

## Abstract

Gastrointestinal (GI) bleeding is an unwanted side effect common to all chemical types of nonsteroidal anti-inflammatory drugs (NSAIDs), particularly in elderly people. However, the risk factors of GI bleeding associated with NSAIDs for elderly people remain unknown. This study aims to evaluate the risks of GI bleeding associated with NSAIDs in 4728 elderly people over 60 years old based on database from a hospital in Beijing.

This retrospective hospital-based study included 4728 patients over 60 years old prescribed with NSAIDs, of which 928 patients had GI bleeding and 3800 did not have. Odds ratios (OR) for the risk of GI bleeding associated with NSAIDs were determined by logistic regression analysis. Mean Decrease Gini (MDG) involved in random forest algorithm was used to rank the associated factors with GI bleeding.

In multivariate analysis, family history of GI bleeding (OR, 3.348; *P = *.000), history of peptic ulcers (OR, 4.068; *P = *.000), history of cardiovascular and cerebrovascular disease (OR, 1.476; *P = *.001), diabetes mellitus (OR, 1.408; *P = *.000), antiplatelet drugs (OR, 3.106; *P = *.000), *Helicobacter pylori* infection (OR, 1.312; *P = *.001), cholesterol level (OR, 0.516; *P = *.000), upper abdominal discomfort (OR, 3.467; *P = *.000), anorexia (OR, 2.038; *P = *.000), and NSAIDs used for 0.5 to 3 months (OR, 0.780; *P = *.000) were associated with GI bleeding. After ranked the MDG of each factor, the top 5 ranked factors associated with GI bleeding were melena, hematemesis, antiplatelet drugs, cholesterol level, and upper abdominal discomfort.

We found that family history of GI bleeding, history of peptic ulcers, history of cardiovascular and cerebrovascular disease, diabetes mellitus, antiplatelet drugs, *Helicobacter pylori* infection, hypocholesterolemia, and NSAIDs used for 0.5 to 3 months were independent risk factors for GI bleeding on people over 60 years old. Meanwhile, upper abdominal discomfort might be the predictor of GI bleeding associated with NSAIDs elderly users.

## Abstract 2

Supplemental Digital Content is available in the text

## 1 Introduction

Nonsteroidal anti-inflammatory drugs (NSAIDs) are a drug class that groups together drugs which provide analgesic and antipyretic effects, and, in higher doses with anti-inflammatory effects. NSAIDs are widely used for various indications, mostly in cardiovascular events and rheumatology. Aging populations are naturally burdened with these diseases. Just because of its antipyretic, analgesic, anti-inflammatory, anti-rheumatic, and anticoagulant effects,^[1,2]^ NSAIDs and low-dose aspirin are commonly prescribed for elderly people in recent decades in China. In the United States, 10% to 20% people above 65 years are treated with NSAIDs^[3,4]^ and many others are self-medicated. A similar level of use has been reported in the Chinese population. To date, aspirin has played an important role in primary and secondary prevention to treat cardiovascular and cerebrovascular diseases, becoming the most widely used NSAID.

Gastrointestinal (GI) bleeding is a severe side effect of NSAIDs which could weaken the defence mechanisms of the GI mucosa and affect hemostasis.^[5–8]^ The GI toxicity of NSAIDs has been explored in a large number of epidemiological studies and clinical trials^.^^[9,10]^ GI mucosa injuries induced by NSAIDs vary from asymptomatic endoscopic erosions/ulcers to ulcer complications (bleeding, perforation, and stenosis). The researchers founded that about 1 out of 100 patients who used aspirin for a mean of 28 months had developed GI bleeding in a meta-analysis of 24 randomized controlled trials.^[11]^ Other researchers also found that among 50,114 incidents with GI bleeding, 5.6% patients were died of taking aspirin or other NSAIDs.^[12]^ Meanwhile, some studies suggested that even low dose of NSAIDs could increase the risk of peptic ulcer by 3 times.^[13,14]^

The prevalence of GI bleeding in elderly patients varies from 2.5% to 4.5%. In most cases, elderly patients suffered from the GI toxicity of NSAIDs may have no significant GI manifestations (abdominal pain, nausea, and vomiting) before bleeding.^[15]^ Due to the analgesic effect of NSAIDs, most clinical symptoms were not typical, so many patients went to hospital to treat GI bleeding before they found themselves already in critical condition.^[16,17]^ In addition, nerve endings are slow to respond for the elderly plagued by diminishing neurological function. Poor regulatory function makes it hard to detect bleeding timely. Symptom monitoring as a supplementary method of disease surveillance provides a solution of early warning, greatly improving the sensitivity of disease diagnosis. If NSAIDs related GI bleeding warning symptoms to elderly people could be clearly identified, doctors can better monitor and prevent GI bleeding in advance. Nevertheless, the predictors of GI bleeding associated with NSAIDs are unknown.

Based on the previous researches, the most relevant risk factors of developing NSAID-induced GI bleeding summarized are:^[18–22]^ age ≥65 years ^[23,24]^ (age >60 years for some scientific associations^[20,25]^); prior occurrence or history of peptic ulcer disease, including previous GI bleeding associated with NSAIDs;^[26]^ and concomitant use of other medications—systemic corticoids,^[23]^ oral anticoagulants,^[27]^ clopidogrel or ticlopidine,^[28]^ alendronate,^[29]^ or other NSAIDs, including low-dose acetylsalicylic acid (80–325 mg/d).^[30]^ Risk assessment is crucial for managing NSAIDs-induced GI damage.^[31]^ So far, some studies have reported the risk factors for and protective strategies against upper GI complications due to NSAIDs usage.^[32,33]^ Although elderly is one of the most important risk factors for NSAIDs-induced GI bleeding, other risk factors of NSAIDs-induced GI bleeding especially for elderly people are poorly understood, which is worthy of further study.

As a result of the current lack of clarity on the subject, we performed a retrospective study to assess the risk factors for GI bleeding in NSAIDs elderly users and to determine the predictors of GI bleeding associated with NSAIDs in elderly patients. The study provided a direct comparison of the risk factors of GI bleeding with nonbleeding in terms of age, gender, past history of PUD, co-morbidity, status of smoking, *Helicobacter pylori* infection, NSAIDs categories, concomitant antiplatelet agent, anticoagulants, glucocorticoids, and GI manifestations from data collected for one decade at Xuanwu Hospital, the largest medical center in the southeast of Beijing, China.

## 2 Methods

### 2.1 Study design, setting, and participants

Based on database from Xuanwu Hospital, a total of 6890 patients were included into this study from the department of gastroenterology, cardiology, neurology, rheumatism, and orthopedics between January 2007 and January 2017 in Xuan Wu Hospital. They were older than 60 years old, prescribed with NSAIDs. Finally, a total of 4728 patients were included for analysis based on the inclusion and exclusion criteria, of which inclusion criteria for case subjects were: ≥ 60 years old; Chinese nationality; taking NSAIDs (for at least 14 days); outpatient onset of GI bleeding and emergently hospitalized at the Xuanwu Hospital between January 2007 and January 2017; having better comprehension and cognitive function; clinically and/or endoscopically verified GI bleeding. Inclusion criteria for control subjects were: ≥ 60 years old; Chinese nationality; taking NSAIDs (for at least 14 days); outpatient and inpatient at the Xuanwu Hospital between January 2007 and January 2017; having better comprehension and cognitive function; clinically and/or endoscopically verified non-GI bleeding. Exclusion criteria for all subjects were: <60 years old; no taking NSAIDs or taking NSAIDs for <15 days; those who were not able to understand questionnaire; those who were not coordinated with the test. All inclusion and exclusion criteria were met before the patients were enrolled. This study protocol was approved by the Institutional Review Board of the Xuanwu Hospital and all patients gave informed consent to the study, which was conducted in accordance with the Ethical & Clinical Assays Committee.

### 2.2 Data sources and assessment

A detailed questionnaire was completed by medical staff during a face-to-face interview with each patient. Medical research staff received unified training on the questionnaire before the test and cross-checked medical records for unanswered questionnaire items to avoid omissions. The diagnosis of GI bleeding was defined as gastric, duodenal, peptic, or gastrojejunal ulcer with hemorrhage or perforation (ICD-10 code K250, K251, K252, K254, K255, K256, K260,K261, K262, K264, K265, K266, K270, K271, K272, K274,K275, K276, K280, K281, K282, K284, K285, K286), acute hemorrhagic gastritis (K290), hematemesis (K920), melena (K921), or unspecified GI hemorrhage (K922). A structured interview covered the following potential risk factors: age, gender, family history of GI bleeding, history of peptic ulcers, NSAIDs categories, history of cardiovascular and cerebrovascular diseases, rheumatoid arthritis, other rheumatism, hematopathy, diabetes mellitus (previous diagnosis, concurrent treatment with insulin or oral hypoglycemic medications, or fasting plasma glucose level >7.0 mmol/L or random blood glucose level >11.1 mmol/L),^[34]^ glucocorticoid, antiplatelet drugs, anticoagulation, medication time, *H pylori* infection, status of smoking (patients who smoked more than 10 cigarettes a day prior to onset for >6 months),^[35]^ cholesterol level (hypocholesterolemia, normal level of cholesterol, and hypercholesteremia), abdominal pain, abdominal distension, upper abdominal discomfort, sour regurgitation, heartburn, belching, anorexia, nausea, vomit, hematemesis, and melena. Patients were asked about their use of the types of NSAIDs, 9 kinds of nonaspirin antiplatelets (ticlopidine, clopidogrel, cilostazol, dipyridamole, sarpogrelate hydrochloride, ethylicosapentate, dilazephydrochloride, limaprostalfadex, and beraprost), and 2 kinds of anticoagulation (warfarin and low molecular heparin). The types of NSAIDs were classified and summarized in all subjects. The primary use of NSAIDs included aspirin, acetaminophen, ibuprofen, indometacin, diclofenac, loxoprofen, meloxicam, oxycodone and paracetamol and celecoxib. The remaining NSAID types were not clearly classified (because of a small proportion) and were unified into a type as “other NSAIDs.” A history of peptic ulcer was defined as a positive answer on the questionnaire or the presence of an ulcer scar on endoscopy. Other rheumatism included systemic lupus erythematosus, Sjogren syndrome, gout et al. Hematopathy included leukaemia, aplastic anemia, myelodysplastic syndrome, thrombocytopenia, multiple myeloma, lymphoma, bones fibrosis, hemophilia, Mediterranean anemia et al. Cardiovascular and cerebrovascular diseases included coronary heart disease, myocardial infarction, cerebral hemorrhage, cerebral thrombosis et al. Hypocholesterolemia refered to TC< 3.24 mmol/L. Hypercholesterolemia was defined as TC >6.2 mmol/L. Normal level of cholesterol referred to 3.24 mmol/L≤TC≤6.2 mmol/L Anorexia refers to no desire of eating or eat significantly less. For the diagnosis of *H pylori* infection, serological testing was used. All people in the 2 groups were tested *H pylori* infection. *H pylori* test was a routine examination for all the people who used NSAIDs in our institution. Medication time groups involved 0.5 to 3 months, 3 to 6 months, 6 to 12 months and over 12 months. Age cohorts included 60 to 69 age group, 70 to 79 age group, and ≥80 age group.

### 2.3 Statistical analysis

Continuous variables were presented with mean ± SD and categorical variables were presented with the number (percentages). Statistical significance of differences was analyzed using independent *t* test or Mann–Whitney *U* test for continuous variables and the chi-square test for categorical variables. Multivariate logistic regression analysis was applied to extract risk factors and calculate odds ratio (OR) with 95% confidence intervals (CI) of NSAIDs on incident GI bleeding events. Mean Decrease Gini (MDG) involved in random forest algorithm was used to rank the associated factors with GI bleeding. MDG provides the ways to quantify which factors contribute most to classification accuracy. Greater MDG will indicate that the degree of impurity arising from category could be reduced farthest by one variable, and thus suggests an important associated factor. Statistical analysis was computed using SPSS 17.0 (SPSS Inc, Chicago, IL) and random Forest package of R software (http://www.r-project.org). All of the statistical tests were 2-sided and considered statistically significant if *P* < .05.

## 3 Results

### 3.1 Demographic data for patients

A total of 928 patients had GI bleeding and 3800 did not have. GI bleeding group over 60 years old were included in this study based on our inclusion and exclusion criteria, in which 556 (59.9%, mean age 72.46 ± 8.12) were male patients, with 372 (40.1%, mean age 72.58 ± 8.29) females. Age range was from 60 to 93 years old, and mean age was 72.50 ± 8.18. 3800 eligible controls (nonbleeding group) over 60 years old were recruited from the same hospital, in which 2239 (58.9%, mean age 70.35 ± 9.21) were male patients, with 1561 (41.1%, mean age 71.04 ± 9.36) females. Age range was from 60 to 95 years old, and mean age was 70.49 ± 9.27. As shown in Table 1, GI bleeding patients had a higher percentage of family history of GI bleeding, history of cardiovascular and cerebrovascular diseases, ibuprofen, indomethacin, diclofenac, loxoprofen, meloxicam, others NSAIDs, hematopathy, diabetes mellitus, glucocorticoid, antiplatelet drugs, *H pylori* infection, status of smoking, hypocholesterolemia, medication time for 0.5to 3 months, medication time for 3 to 6 months, abdominal pain, upper abdominal discomfort, sour regurgitation, belching, anorexia, nausea, hematemesis, and melena than those patients without GI bleeding. Meanwhile, GI bleeding patients had a lower percentage of aspirin, acetaminophen, oxycodone and paracetamol, celecoxib, rheumatoid arthritis, other rheumatism, anticoagulation, normal level of cholesterol, hypercholesteremia, medication time for 6 to 12 months, medication time for >12 months, heartburn and vomit than those patients without GI bleeding. The same percentage of abdominal distension occurred between the 2 groups. No significant differences between-group in age (*Z* = 0.844, *P* = .399) and age groups (χ2 = 3.108, *P* = .211) were found.

**Table 1:**
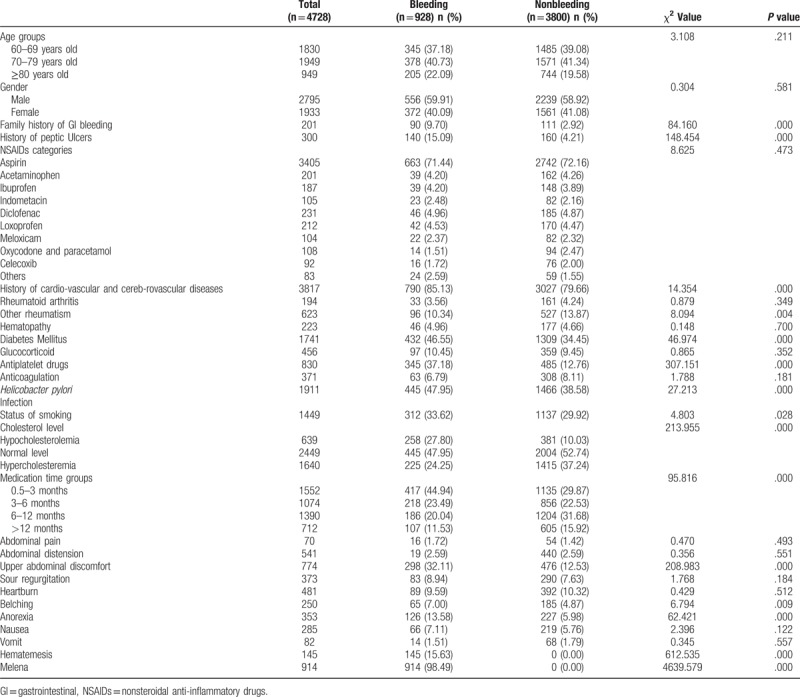
Comparisons risk factors of GI bleeding associated with NSAIDs drugs between groups.

### 3.2 Risk factors of GI bleeding associated with NSAIDs drugs between groups

All 4728 subjects were separated into 2 groups based on GI bleeding findings. As shown in Table 1, compared to subjects without GI bleeding, more patients with GI bleeding had family history of GI bleeding and history of peptic ulcers. GI group had more patients with history of cardiovascular and cerebrovascular diseases, other rheumatism and diabetes mellitus (*P* < .05). Furthermore, proportions of patients with concomitant antiplatelet drugs, *H pylori* infection and status of smoking were also considerably higher in GI bleeding group compared to non-GI bleeding group (*P < *.05). Also patients with GI bleeding had significantly hypocholesterolemia (*P < *.05, for all comparison). NSAIDs medication time was compared between bleeding group and nonbleeding group, and there was statistical difference (*Z* = 8.371, P = .000). Comparisons among different medication time groups indicated: patients taking medicine for 0.5 to 3 months were most vulnerable to GI bleeding. Although NSAIDs-induced GI bleeding was featured with hematemesis and /or melena as the first symptom, this study found that a higher proportion of patients with upper abdominal discomfort (*P = *.000), anorexia (*P = *.000) and belching (*P = *.009) in bleeding group.

### 3.3 Logistic regression analysis for risk factors of GI bleeding associated with NSAIDs drugs

Logistic regression analysis was performed to evaluate independent risk factors (mentioned in Table 1) for GI bleeding accidents in patients with NSAIDs. As shown in Table 2, risk factors including family history of GI bleeding, history of peptic ulcers, history of cardiovascular and cerebrovascular disease, diabetes mellitus, antiplatelet drugs, *H pylori* infection, hypocholesterolemia, upper abdominal discomfort, anorexia, and NSAIDs used for 0.5 to 3 months increased the risk of elderly GI bleeding associated with NSAIDs drugs (*P < *.05).

**Table 2:**
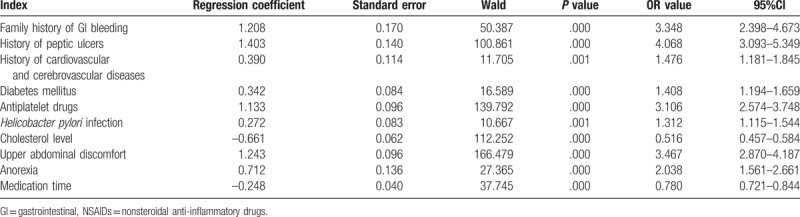
Multivariate logistic regression analysis of independent risk factors for GI bleeding accidents in patients with NSAIDs.

### 3.4 Random forest algorithm to rank associated factors with GI bleeding

**Figure 1:**
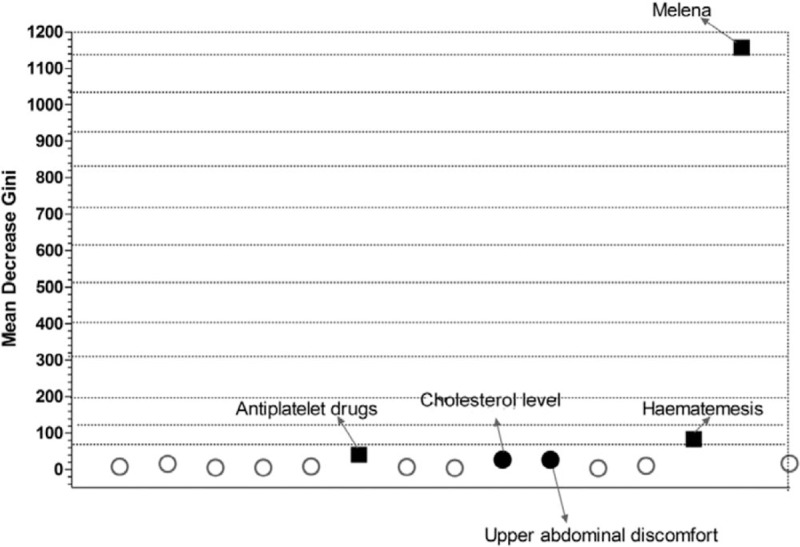
Result of the random forest model for evaluation the risk factors for NSAIDs-induced GI bleeding. Thirteen potential factors associated with GI bleeding based on univariate analysis, hematemesis and melena came into the random forest model. After ranked the MDG of each factor, the top 5 ranked factors associated with GI bleeding were melena, hematemesis, antiplatelet drugs, cholesterol level, and upper abdominal discomfort. GI = gastrointestinal, MDG = Mean Decrease Gini, NSAIDs = nonsteroidal anti-inflammatory drugs.

Around 13 potential factors associated with GI bleeding based on univariate analysis, hematemesis and melena came into the random forest model. These 13 factors included family history of GI bleeding, history of peptic ulcers, history of cardiovascular and cerebrovascular disease, other rheumatism, diabetes mellitus, antiplatelet drugs, *H pylori* infection, status of smoking, cholesterol level, medication time (months), upper abdominal discomfort, belching, and anorexia. After ranked the MDG of each factor, the top 5 ranked factors associated with GI bleeding were melena, hematemesis, antiplatelet drugs, cholesterol level, and upper abdominal discomfort (see Table 3 and Fig. 1).

**Table 3:**
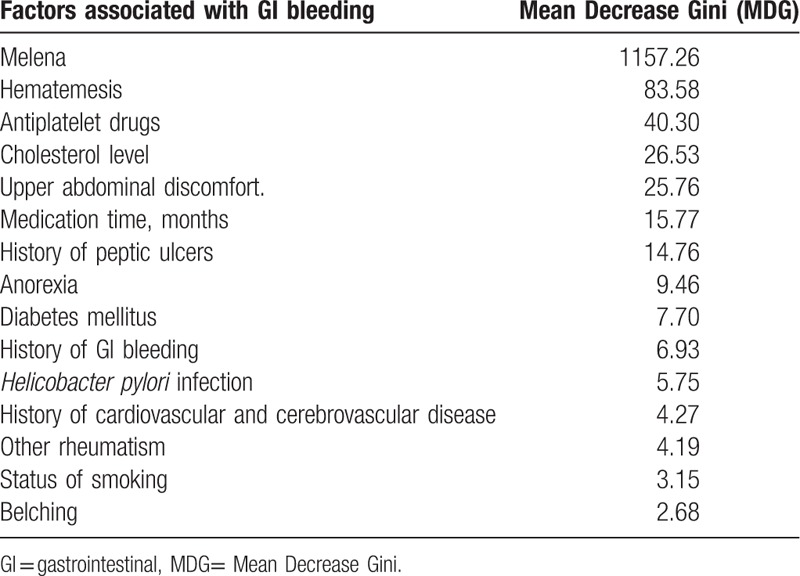
The rank for factors associated with GI bleeding based on MDG.

**Figure 2:**
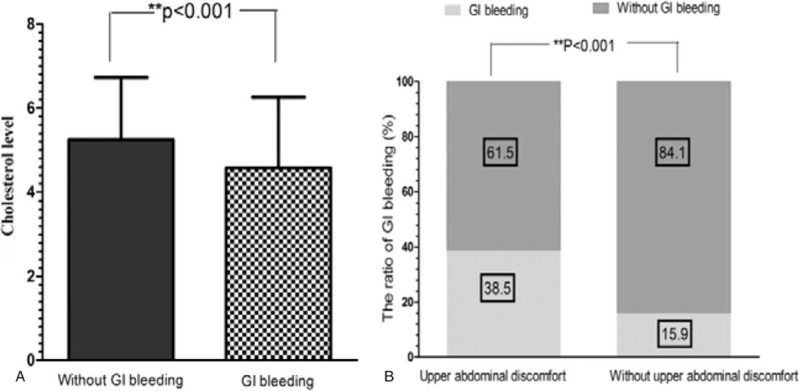
The cholesterol level and upper abdominal discomfort were the identified novel risk factors associated with GI bleeding. The cholesterol level of GI bleeding was lower than those without GI bleeding (*P < *.001, [A]). The proportion of patients with upper abdominal discomfort with GI bleeding (38.5%) was higher than the proportion without GI bleeding (15.9%) (*P < *.001, [B]). GI = gastrointestinal.

Notably, we found that cholesterol level and upper abdominal discomfort were the identified novel risk factors associated with GI bleeding. We observed that the cholesterol level of GI bleeding was lower than those without GI bleeding (Fig. 2A). Compare with those patients without upper abdominal discomfort, a higher proportion of patients with upper abdominal discomfort were found to occur GI bleeding (Fig. 2B).

## 4 Discussion

This study did not find the factor of age to have a significant impact on NSAIDs-related GI bleeding because all of the 4728 subjects were elderly patients (age ≥60 years). And there was no significant difference between 3 different age groups. It was not found the relative increases in risk of GI bleeding among NSAIDs users in very elderly patients (defined as >80 years). These observations were consistent with a meta-analysis of 18 studies.^[36]^ We found that the history of peptic ulcer disease, family history of GI bleeding, and concomitant use of antiplatelet drugs were independent risk factors for elderly patients with NSAIDs, on the contrary, concomitant use of corticosteroids and anticoagulants was not independent factors in this study. It is inconsistent with the American College of Gastroenterology Guidelines for Prevention of NSAID-Related Ulcer Complications (ACG Guideline) which regards concomitant use of corticosteroids and anticoagulant with NSAIDs as indicators of NSAID-induced gastrointestinal damages.^[37]^ This could be explained by application of gastroprotective drugs (proton pump inhibitor, PPI, or H2 blocker)^[38]^ Patients (complication of rheumatoid arthritis and other rheumatic disease) used NSAIDs and glucocorticoids, a part of which used gastroprotective drugs concurrently. It might reduce the incidence of GI bleeding caused by NSAIDs. The gastroprotection was not included in the analysis as an associated factor with GI bleeding, because only a small group of patients had used it. Many previous studies suggested that fairly wide variation in GI bleeding rates among different NSAIDs, with ketoprofen and piroxicam generally associated with higher and ibuprofen with lower risks.^[39–44]^ Rafaniello et al^[45]^ pointed that the highest association with GI events was observed for ketorolac exposure followed by nimesulide, diclofenac, aspirin, ketopro-fen, and ibuprofen among individual NSAIDs, and also found an higher positive association for NSAID monotherapy than for aspirin alone. Nevertheless, in our study, we did not find that different types of NSAIDs were independent risk factors for elderly patients which were not in accordance with previous studies estimating the risk of upper or lower GI complications of NSAIDs, aspirin and their combination.^[46,47]^

In this study, most of the risk factors were complied with previous research findings.^[48–50]^ We paid particular attention to factors of diabetes mellitus and hypocholesterolemia. Diabetes is a well-known independent risk factor for peptic ulcer bleeding, contributing a population attributable fraction of 4%.^[26]^ Studies by Lin et al^[51]^ indicated that among the test group who took low dose aspirin as secondary prevention measure for cardiovascular diseases, patients with diabetes with *H pylori* infection were 4 times likely to suffer from GI bleeding than others. Furthermore, Kim et al^[52]^ found that elderly patients with diabetes treating NSAIDs had a significantly higher risk of upper GI bleeding compared to NSAID nonusers. This study also indicated that diabetes was likely to increase NSAIDs GI bleeding risk for elderly people (OR = 1.408, 95%CI 1.194–1.659). Therefore, it is important to monitor the incidence of GI bleeding in elderly people with diabetes when using NSAIDs. Studies published in recent years found abnormal blood lipid level was closely associated with visceral hemorrhage. Greater lipid level deviance generated higher risk of bleeding. Previous studies had demonstrated a clear relation between low level lipid and cerebral hemorrhage,^[53]^ but there has yet to be enough attention to the relations between lipid level and GI bleeding. This study indicated that hypocholesterolemia was a risk factor for NSAIDs related GI bleeding for elderly people. Recent reports from researchers also indicated negative correlation between cholesteremia and GI bleeding,^[54,55]^ which means lower serum cholesterol gives rise to GI bleeding risk while prognosis of patients were getting worse. Feeding animals with insufficient cholesterol could lead to higher fragility of red blood cells and cause them to burst. Low cholesterol levels may cause walls of large arteries to become fragile with reduced tenacity and elasticity, followed by vessel complications and then bleeding.^[56]^However, no clear mechanism has been defined, except for presumption that cholesterol engages in multiple organ failures.

Current European and US guidelines recommend test and treat *H pylori* infection in NSAIDs users who are at risk of upper GI bleeding.^[57–59]^ Despite these guidelines, whether NSAIDs use and *H pylori* infection may interact to increase the risk of GI bleeding in the elderly is uncertain.^[60]^ Our study suggested that *H pylori* infection was a risk factor for elderly patients with NSAIDs related GI bleeding (OR = 1.312, 95% CI 1.115–1.544). In accordance with the following studies,^[61]^ NSAIDs use and *H pylori* infection are important risk factors for GI bleeding with a synergistic effect. Some studies support that the long-term incidence of GI bleeding with NSAIDs use is low after *H pylori* eradication alone despite a history of ulcer bleeding. Eradication of *H pylori* in elderly patients before starting NSAIDs therapy may be an effective policy in preventing GI bleeding. Nevertheless, some previous researches about elderly patients had opposite findings.^[62]^ Meanwhile, other report demonstrated NSAIDs intake was more frequent in patients with upper GI bleeding (34%) than in those without upper GI bleeding (5.6%), while *H pylori* infection was less frequent in patients with upper GI bleeding [92.4% (85–96%)] than in those without upper GI bleeding [99.1% (98–100%); *P* < .001]. In another 12-month randomized trial, however, up to 15% of aspirin users developed recurrent ulcer bleeding after eradication of *H pylori* alone^.^^[63]^ In light of these conflicting findings, the long-term benefit of eradicating *H pylori* in high-risk NSAIDs users is uncertain. Nevertheless, current guidelines recommend that the treatment of *H pylori* positive patients with GI bleeding using eradication therapy is effective and co-therapy with a PPI is still needed in high-risk NSAIDs users after eradication of *H pylori*.

Because symptom monitoring as a supplementary method of disease surveillance provides a solution of early warning of GI bleeding, this study analyzed the symptoms of digestive system and discovered that upper abdominal discomfort and loss of appetite were significantly higher in bleeding group than the other, it is more obvious with upper abdominal discomfort. Upper abdominal discomfort may be warning symptoms of NSAIDs related GI bleeding for the elderly. Giving attention to these 2 symptoms, doctors and patients may be able to “predict” the occurrence of GI bleeding to some extent before applying early screening and protective measures (such as: the application of PPI preparation), thereby effectively improving the prognosis of patients.

Moreover, a recent literature^[64]^ provided a set of guidelines which help to enable correct application of machine learning algorithms. A prediction model based on machine learning algorithms is usually constructed based on the training dataset and is evaluated using the testing dataset. In order to avoid the problems like over-fitting, we additionally adopt 5-fold cross-validation to implement the analysis. That is, the whole samples were divided into 5 sets. For each analysis, one set was considered as testing data whereas the remaining sets were considered as training data. We found the classification accuracy rate were all up to 100.0% (see Fig. 1, Supplemental Content;, which illustrates the classification accuracy rate were all up to 100.0%). In addition, for each analysis, we found the top 3 ranked factors associated with GI bleeding were melena, hematemesis, and antiplatelet drugs according to MDG involved in random forest algorithm (see Table 1, Supplemental Content;, which illustrates the top 3 ranked factors associated with GI bleeding were melena, hematemesis, and antiplatelet drugs in random forest algorithm). These results provide further support to our findings. We also provided the R code of our analysis (see Table 2, Supplemental Content;, which illustrates the R code of the analysis). In particular, we kept the constructed random forest model based on the whole sample and prepare to validate this model using external data in future studies.

Despite the fact that NSAIDs may cause GI bleeding and a series of adverse reactions, the drugs are irreplaceable in terms of clinical value to elderly patients. How to use this “double-edged sword” of NSAIDs and how to correctly assess its “risk and benefits” to patients remains a key research topic.^[65,66]^ It has been found that close monitoring and assessing various risk factors during NSAIDs medication is one of the ways to effectively reduce GI bleeding to the elderly. We analyzed the relevant risk factors for elderly patients and revealed some individual factors not mentioned in previous studies. Nevertheless, the downside of this study was that it was a retrospective study. A series of prospective studies are required to support the bleeding-related risk factors found in this study, which is exactly what we are working on.

## 5 Conclusions

In conclusion, family history of GI bleeding, history of peptic ulcers, history of cardiovascular and cerebrovascular disease, diabetes mellitus, antiplatelet drugs, *H pylori* infection, hypocholesterolemia, and NSAIDs used for 0.5 to 3 months were independent risk factors for GI bleeding on people over 60 years old. Meanwhile, upper abdominal discomfort seems to be the predictor of GI bleeding associated with NSAIDs elderly users.

## Author contributions

**Conceptualization:** Tian-Yu Chi, Mei Zhang.

**Data curation:** Tian-Yu Chi, Hong-Ming Zhu.

**Formal analysis:** Tian-Yu Chi, Mei Zhang.

**Funding acquisition:** Mei Zhang.

**Investigation:** Tian-Yu Chi, Hong-Ming Zhu.

**Methodology:** Tian-Yu Chi.

**Project administration:** Tian-Yu Chi, Mei Zhang.

**Resources:** Tian-Yu Chi.

**Software:** Tian-Yu Chi.

**Supervision:** Tian-Yu Chi, Mei Zhang.

**Visualization:** Tian-Yu Chi.

**Writing – original draft:** Tian-Yu Chi.

**Writing – review & editing:** Tian-Yu Chi, Mei Zhang.

## Supplementary Material

Supplemental Digital Content
