# Supplementary material for: Risk factors associated with nonsteroidal anti-inflammatory drugs (NSAIDs)-induced gastrointestinal bleeding resulting on people over 60 years old in Beijing
Source: Medicine (Baltimore). 2018 May 4;97(18):e0665. doi: 10.1097/MD.0000000000010665 (PMC6392961; doi:10.1097/MD.0000000000010665)
Supplement: Supplemental Digital Content [file medi-97-e0665-s001.docx]

Supplementary Figure 1 5-fold cross validation process for random forest program


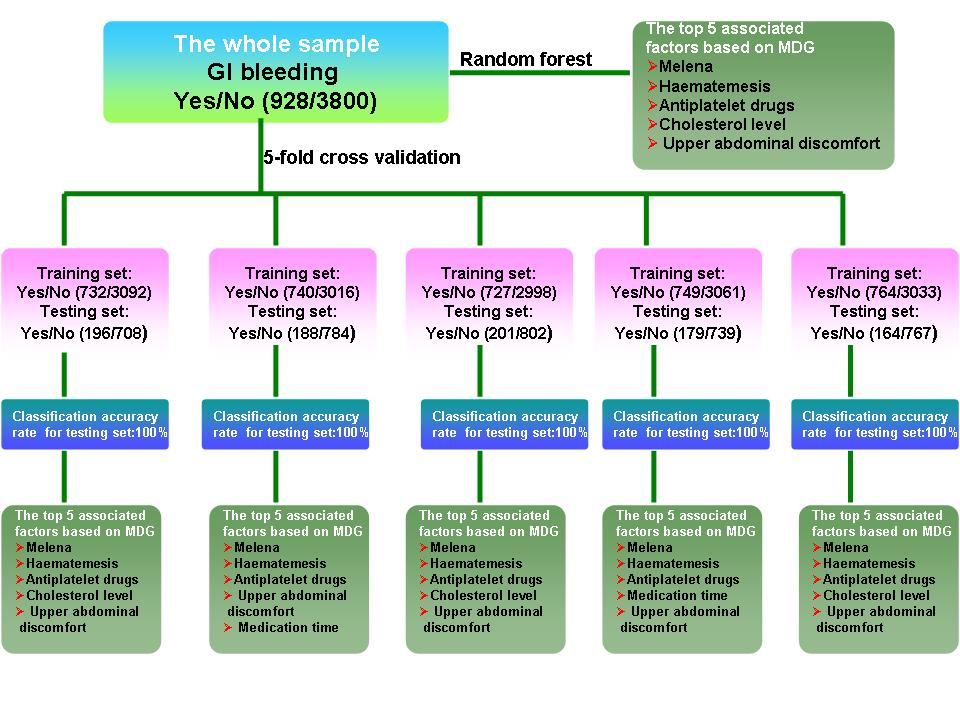


Supplementary Table 1 The rank of MDG based on 5-fold cross validation

|  | The rank of Mean Decrease Gini (MDG) | | | | |
| --- | --- | --- | --- | --- | --- |
|  | 1^st^-fold | 2^nd^-fold | 3^rd^-fold | 4^th^-fold | 5^th^-fold |
| Melena | 1 | 1 | 1 | 1 | 1 |
| Haematemesis | 2 | 2 | 2 | 2 | 2 |
| Antiplatelet drugs | 3 | 3 | 3 | 3 | 3 |
| Cholesterol level | 4 | 6 | 4 | 6 | 4 |
| Upper abdominal discomfort. | 5 | 4 | 5 | 5 | 5 |
| Medication time(months) | 6 | 5 | 6 | 4 | 6 |
| History of peptic ulcers | 7 | 7 | 7 | 7 | 7 |
| Anorexia | 9 | 8 | 8 | 8 | 9 |
| Diabetes mellitus | 10 | 9 | 10 | 9 | 10 |
| History of GI bleeding | 8 | 10 | 9 | 11 | 8 |
| *Helicobacter pylori* infection | 11 | 11 | 11 | 10 | 11 |
| History of cardiovascular and cerebrovascular disease | 12 | 12 | 13 | 12 | 13 |
| Other rheumatism | 13 | 13 | 12 | 13 | 12 |
| Status of smoking | 14 | 14 | 14 | 14 | 14 |
| Belching | 15 | 15 | 15 | 15 | 15 |

Supplementary Table 2 R code of random forest analysis in our study

| library(randomForest)  read.table("c:\\randomforest.csv",header=TRUE,sep=",")->a  data<-a  k=5  id<-sample(1:k,nrow(data),replace=TRUE)  list<-1:k  train<-subset(data, id %in% list[-1])  for (i in 1:k){train<-subset(data, id %in% list[-i])  test<-subset(data,id %in% c(i))  model<-randomForest(group~.,data=train, importance=TRUE)  print(importance(model, type=2))  pred<-predict(model,test[,-16],type="class")  n<-test[,"group"]  print (table(pred,n))  } |
| --- |
